# Function-preserving intracapsular versus superficial parotidectomy for benign parotid tumors: A randomized controlled trial

**DOI:** 10.5339/qmj.2026.51

**Published:** 2026-08-11

**Authors:** Ayman Abdulmohaymen, Sayed R. Abdelbary, Tamer A. Abouelgreed, Abd El-Fattah Al Sheikh, Hany Abdelfatah Elhady, Zahraa Futooh, Mahmoud E. Nagaty, Hamada Rashad Abdelkader, Ahmed F. Elhossainy, Mohamed I. Shalamesh, Alsayed B. Moghazy, Mohamed A. Nafea, Hamdy Farag, Esam M. Osman, Mohammed Abbas, Islam M. Ibrahim, Mohamed Hassan Elkaseer, Mohamed Mahdy, Mahmoud A. El-Hussieny, Ahmed G. E. Metwally, Mohammed M. Elrefaey, Ahmed Lamey

**Affiliations:** 1 Department of Surgical Oncology, Faculty of Medicine, Al-Azhar University, Cairo, Egypt; 2 Department of Urology, Faculty of Medicine, Al-Azhar University, Cairo, Egypt; 3 Department of General Surgery, Faculty of Medicine, Al-Azhar University, Cairo, Egypt; 4 Department of Surgery, Faculty of Medicine, Jouf University, KSA; 5 Department of General Surgery, Faculty of Medicine, Al-Azhar University, Asyut, Egypt; 6 Department of General Surgery, Faculty of Medicine, Kafr Elsheikh University, Egypt

**Keywords:** Benign parotid tumor, intracapsular parotidectomy, superficial parotidectomy, facial nerve, pleomorphic adenoma.

## Abstract

**Background:**

Superficial parotidectomy is the traditional treatment for benign parotid tumors but may be associated with facial nerve dysfunction and other morbidities. Intracapsular parotidectomy has emerged as a function-preserving alternative; however, concerns regarding oncologic safety remain. Randomized evidence comparing both techniques is limited. Therefore, this study aimed to compare their functional and oncologic outcomes.

**Objective:**

This study aimed to compare intracapsular parotidectomy and superficial parotidectomy in terms of operative efficiency, complications, facial nerve preservation, and tumor recurrence for benign parotid tumors.

**Methodology:**

This multicenter randomized controlled trial included patients with benign parotid tumors who were randomly assigned to undergo either intracapsular or superficial parotidectomy between May 2019 and May 2023, in Cairo, Egypt. Functional outcomes, postoperative complications, and recurrence rates were evaluated.

**Results:**

The mean age was 46.3 ± 9.5 years, with a range of 25–65 years. Most patients (84, 70%) were male, while 36 (30%) were female. The intracapsular parotidectomy (ICP) group demonstrated significantly reduced operative time (68.5 ± 12.3 vs. 88.3 ± 14.5 minutes; *p* = 0.01) and blood loss (120 ± 55.3 vs. 170 ± 50.5 ml; *p* = 0.001) compared to the superficial parotidectomy (SP) group. Facial nerve injury occurred exclusively in the SP group 9 (15%), including 3 (5%) cases of permanent palsy. Recurrence was observed in 1 (1.7%) ICP case and 3 (5%) SP cases, with no statistically significant difference (*P* > 0.05). Other complications, including seroma formation and postoperative bleeding, were similarly distributed between the two groups.

**Conclusion:**

Intracapsular parotidectomy is a safe, effective, and less invasive alternative to superficial parotidectomy for benign parotid tumors. It offers shorter operative time, reduced bleeding, and improved facial nerve preservation without compromising oncological outcomes. Careful patient selection is essential for optimal results.

## 1. INTRODUCTION

Parotid gland tumors represent a major subset of salivary gland neoplasms, with benign lesions accounting for 70–80% of cases. Pleomorphic adenomas are the most common, followed by Warthin tumors, typically affecting adults in midlife and showing a female predominance. Surgical excision is the mainstay of treatment, aiming for complete resection with preservation of facial nerve function.^1,2^ Superficial parotidectomy (SP), the traditional approach, involves facial nerve dissection and removal of the superficial lobe. Though effective, it carries risks like nerve dysfunction, Frey’s syndrome, and cosmetic concerns.^3,4^ To reduce these complications, less invasive techniques, such as intracapsular parotidectomy (ICP), have been developed. ICP excises the tumor along its capsule without dissecting the facial nerve, thereby minimizing trauma and preserving glandular tissue.^3,4^ ICP is recommended for small, mobile, and superficially located benign tumors with low suspicion of malignancy. However, concerns remain regarding capsular rupture and recurrence, particularly in pleomorphic adenomas with microscopic extensions^5,6^ Unlike SP, ICP avoids direct facial nerve manipulation, reducing the risk of transient palsy.^7^ In selected cases, recurrence rates are reported to be low and comparable to SP when performed with meticulous technique^8^ ICP also offers better cosmetic outcomes, less postoperative discomfort, and improved patient satisfaction due to its minimally invasive nature.^4^ It may further reduce operative time, need for nerve monitoring, and hospital stay, contributing to greater cost-efficiency.^9^ Despite its advantages, the adoption of ICP remains inconsistent due to limited long-term data and confusion with related techniques like extracapsular dissection.^7^ Although several studies have compared intracapsular and superficial parotidectomy, most are retrospective or non-randomized and originate from single institutions, with variable outcome measures and limited high-level evidence. Consequently, uncertainty persists regarding the balance between functional preservation and oncologic safety. To address this gap, we conducted a multicenter randomized controlled trial to provide robust comparative data on postoperative facial nerve function, complications, and recurrence rates between the two surgical techniques.

## 2. METHODOLOGY 

This prospective, multicenter, randomized study was conducted in Bab-El-Shaareya University Hospital, Al-Hussain Hospital, and Al-Zahraa Hospital in Cairo, Egypt, between May 2019 and May 2023. This study was conducted in accordance with the principles of the Declaration of Helsinki. Ethical approval was obtained from the Institutional Research Ethics Committee (Approval No. surg.onc.043/35) in April 2019 prior to patient enrollment. The study was registered at ClinicalTrials.gov (Registration No. NCT07185542). Written informed consent was obtained from all participants before inclusion in the study. Patients were randomly assigned in a 1:1 ratio into two groups using a computer-generated randomization sequence. Group 1 underwent intracapsular parotidectomy, while Group 2 underwent superficial parotidectomy. Allocation concealment was maintained using sealed opaque envelopes opened just before surgery. The inclusion criteria were: (1) age between 18 and 65 years, (2) radiologically and clinically confirmed benign parotid tumor, (3) tumor confined to the superficial lobe without evidence of facial nerve involvement, and (4) tumor size less than 4 cm. The exclusion criteria were: (1) suspicion of malignancy based on imaging or fine-needle aspiration cytology (FNAC), (2) recurrent or deep parotid tumors, (3) previous parotid surgery, (4) facial nerve dysfunction preoperatively, (5) comorbidities precluding surgery, and (6) large parotid tumor more than 4cm. All patients underwent detailed history taking and clinical examination. Fine-needle aspiration cytology (FNAC) was done for every patient for preoperative tissue diagnosis. Ultrasound of the parotid region was done as the baseline imaging. Contrast-enhanced computed tomography (CT) or magnetic resonance imaging (MRI) was done in selected cases based on tumor size, depth, or suspicion of deep lobe extension. Tumor size and mobility were documented. Comorbid conditions, such as diabetes mellitus and hypertension, were also recorded.

Due to the nature of surgical interventions, blinding of the operating surgeons was not feasible. However, several measures were taken to minimize bias, such as randomization, where patients were randomly assigned to either intracapsular or superficial parotidectomy using a computer-generated sequence, and postoperative functional outcomes and complications assessment by independent clinicians who were blinded to the type of surgery performed.

All procedures were performed by three experienced head and neck surgeons across the participating centers. Each surgeon performed cases in both the intracapsular and superficial parotidectomy groups to minimize inter-surgeon variability. All participating surgeons had substantial prior experience (>50 parotidectomies each) with both techniques. No surgeon was in the learning phase during the study, and standardized operative protocols were followed to ensure consistency and reproducibility across centers. The sample size of 120 patients was primarily determined based on feasibility, including the study timeframe and patient recruitment capacity. This number is consistent with sample sizes reported in similar studies in the literature. Furthermore, we ensured that this sample was sufficient to provide a reasonable level of precision in estimating the outcomes and to perform the planned statistical analyses, while also allowing for potential loss to follow-up.

### 2.1. Surgical techniques

**Figure 1.**
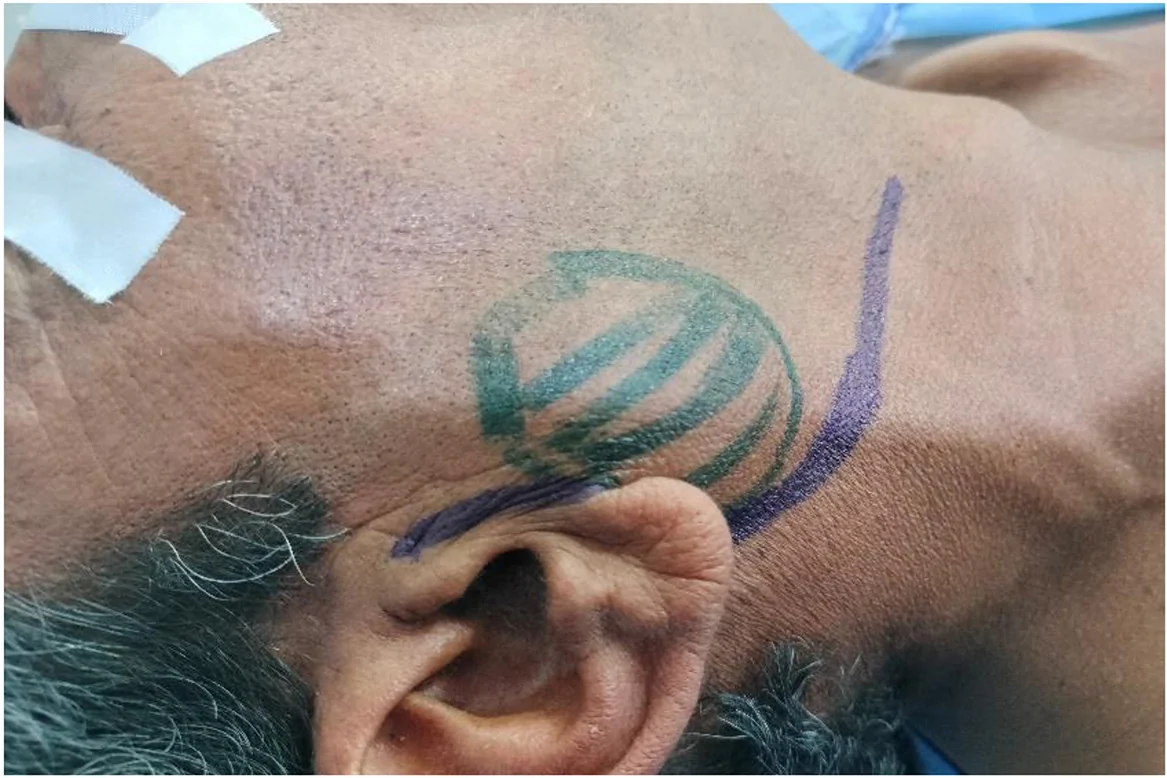
Patient position and marking of the lesion and incision.

**Figure 2.**
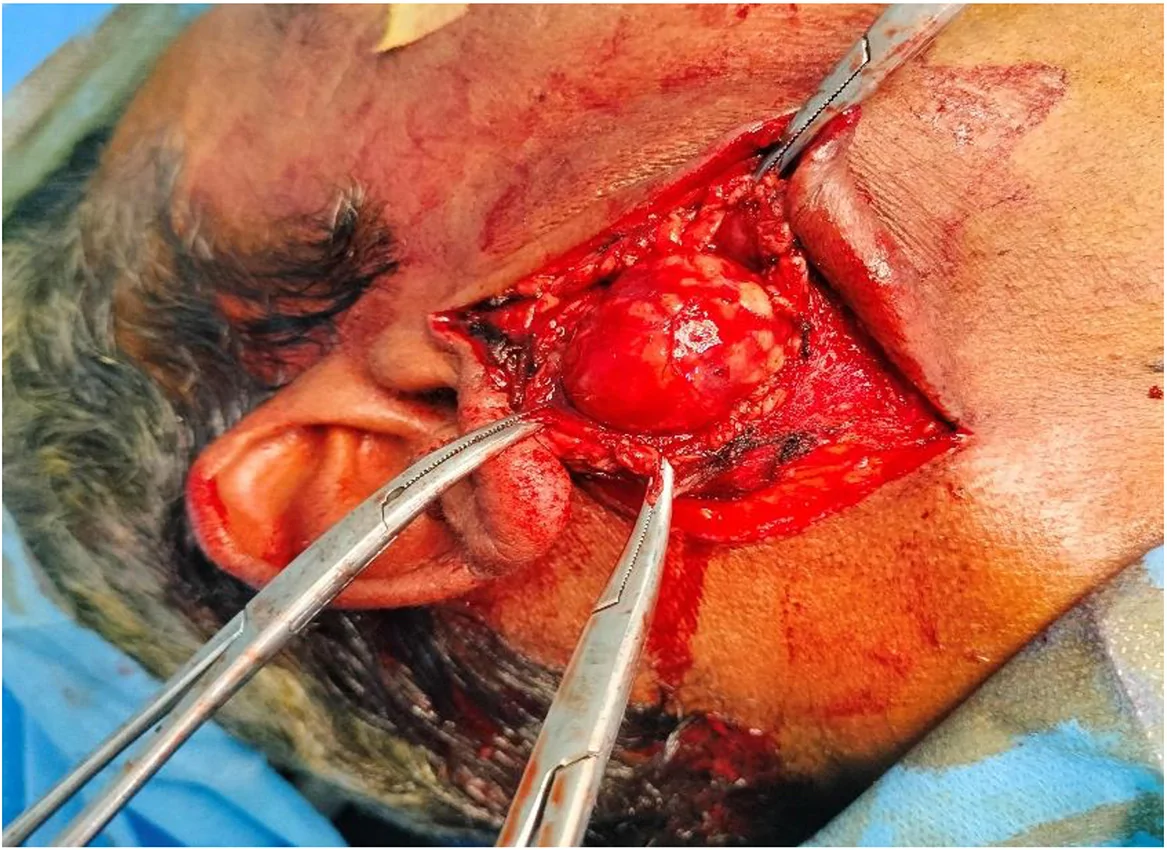
Opening the parotid capsule and identification of the lesion.

**Figure 3.**
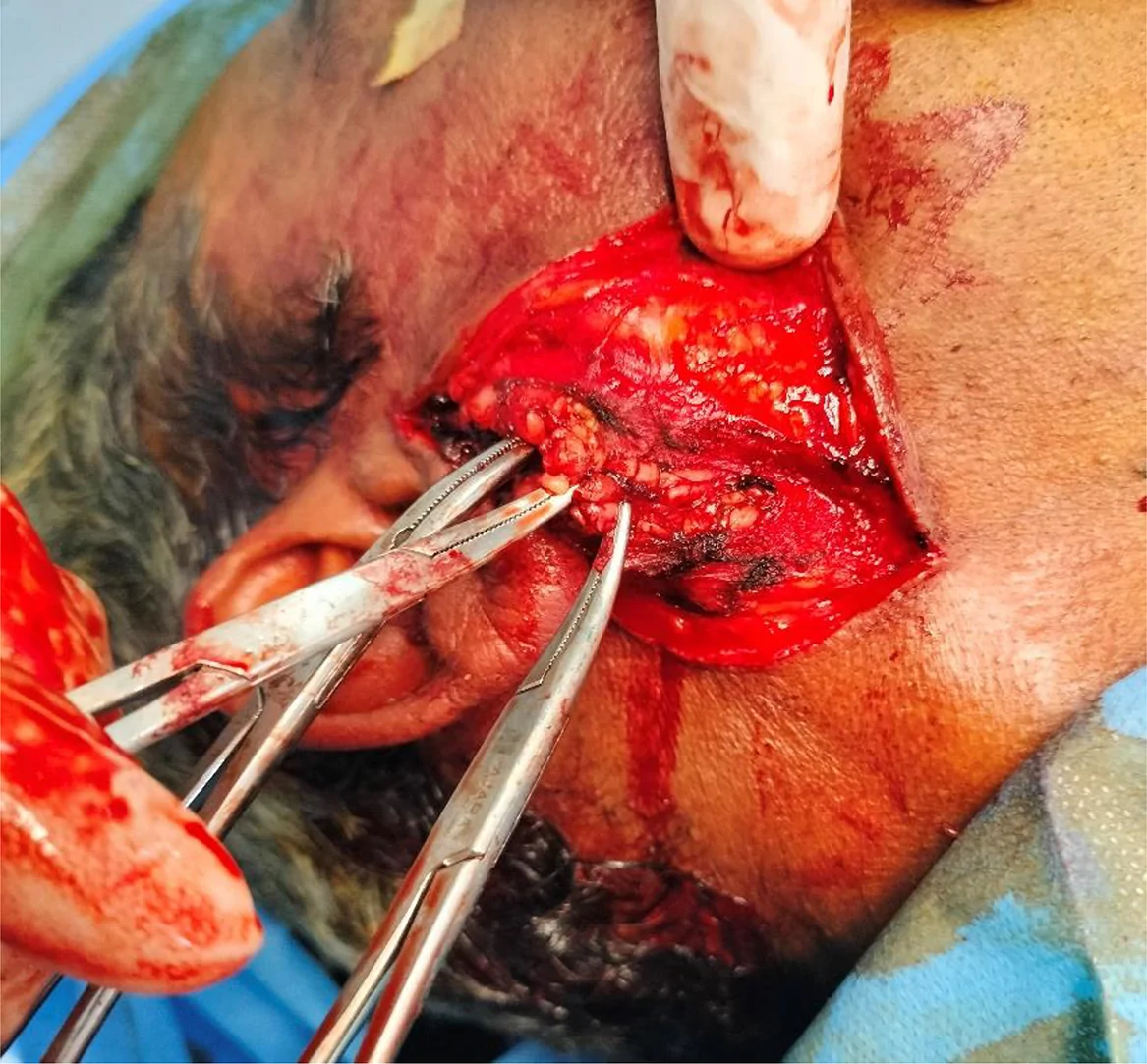
Marking the closure of the parotid capsule.

**Group 1−Intracapsular parotidectomy:** All patients in the ICP group were positioned supine with the head turned to the contralateral side and the neck slightly extended to optimize exposure of the parotid region. After proper antiseptic skin preparation and draping, a modified Blair’s incision was marked along a natural skin crease, extending from the preauricular region down to the cervical area, following the anterior border of the sternocleidomastoid muscle (Figure 1). Dissection proceeded through the subcutaneous tissue and superficial musculoaponeurotic system (SMAS) to expose the parotid fascia. The parotid capsule was then carefully opened (Figure 2), and the tumor was identified through gentle blunt dissection at the margin of its pseudocapsule. The main trunk of the facial nerve was not routinely identified, although the surgeon maintained awareness of its expected anatomical course to minimize inadvertent injury. Once the lesion was adequately exposed, it was excised by performing a careful extracapsular dissection along the tumor’s natural cleavage plane. Special attention was paid to avoid capsular rupture, and any tumor-adjacent parotid tissue was preserved where oncologically safe. Hemostasis was achieved using bipolar cautery, and the surgical field was irrigated (Figure 2). After tumor removal, the parotid capsule was approximated using absorbable sutures to restore anatomical integrity (Figure 3). Layered wound closure was completed using absorbable sutures for deeper layers and subcuticular sutures for the skin. No facial nerve monitoring was used routinely, as direct nerve dissection was avoided in this technique.^10^

**Group 2−Superficial parotidectomy:** Patients in the SP group were positioned similarly to the ICP group. A standard modified Blair’s incision was marked to provide wide access to the parotid region. After incision through skin and SMAS, the parotid fascia was incised to expose the superficial lobe of the parotid gland. Unlike the ICP approach, SP involved meticulous identification of the main trunk of the facial nerve at its emergence from the stylomastoid foramen. This was achieved by tracing anatomical landmarks, such as the posterior belly of the digastric muscle and the tympanomastoid suture. Once the nerve trunk was visualized, careful dissection was carried out along its branches to allow mobilization of the superficial lobe. The tumor-bearing portion of the gland was excised en bloc with surrounding glandular tissue to ensure complete removal. Facial nerve branches were preserved during dissection but were inevitably manipulated, which contributed to a higher incidence of transient nerve dysfunction. Hemostasis was ensured, and the surgical field was inspected before closure. A closed suction drain was placed, and the wound was closed in layers as described above. Facial nerve monitoring was selectively used in cases with difficult dissection or proximity of the tumor to the nerve.^11^

### 2.2. Postoperative assessment

Operative time and intraoperative blood loss were recorded. Patients were monitored for complications, including seroma, hematoma, facial nerve injury (temporary or permanent), and Frey’s syndrome. Histopathological analysis of the resected specimens confirmed tumor type and margin status.

### 2.3. Primary and secondary outcomes

Primary outcomes were the incidence of postoperative facial nerve dysfunction (temporary or permanent) and recurrence rate. Secondary outcomes were operative time, intraoperative blood loss, and incidence of Frey’s syndrome, hematoma, and seroma.

### 2.4. Follow-up

Patients were followed for 2 years postoperatively to assess recurrence, which was defined as radiological or clinical evidence of tumor reappearance at the surgical site.

### 2.5. Statistical analysis

Statistical analysis was performed with Statistical Package for the Social Sciences (SPSS) software, version 26 (IBM, Chicago, Illinois, USA). The normality of the data was tested by the Kolmogorov-Smirnov test. Qualitative data were presented as numbers and percentages and were compared by the Chi-square test or Fisher’s exact test. Quantitative data were presented as mean and standard deviations and were compared by the independent Student’s t-test. As a result, the p-value was considered significant at the level of < 0.05.

## 3. RESULTS

A total of 120 patients were included in our study. The mean age was 46.3 ± 9.5 years, with a range of 25–65 years. Most patients, 84 (70%), were male, while 36 (30%) were female. The two groups were comparable in terms of their demographics (*p* > 0.05 for both age and gender). The mean tumor size was 2.7 ± 0.8 cm in Group 1 and 2.9 ± 1.0 cm in Group 2, with no statistically significant difference between the two groups (*p* = 0.31). The mean duration of symptoms was relatively similar in both groups: 14.9 ± 5.8 months in Group 1 and 15.5 ± 7.0 months in Group 2 (*p* = 0.72). With regard to comorbidities, 25% of the patients in Group 1 were diabetic versus 35% in Group 2, and 10% in Group 1 were hypertensive compared to 35% in Group 2 (*p* = 0.3) (Table 1).

Intraoperatively, the mean operative time was significantly lower in Group 1 (68.5 ± 12.3 minutes) than in Group 2 (88.3 ± 14.5 minutes) (*p* = 0.01), suggesting that the intracapsular approach may offer superior time efficiency compared to the superficial technique. Similarly, the mean intraoperative blood loss was significantly less in Group 1 (120 ± 55.3 ml) than in Group 2 (170 ± 50.5 ml) (*p* = 0.001), indicating better hemostasis and potentially fewer complications with the intracapsular technique (Table 2).

Regarding histopathological findings, 42 (70%) tumors in Group 1 and 36 (60%) in Group 2 were pleomorphic adenomas, while 18 (30%) in Group 1 and 24 (40%) in Group 2 were Warthin tumors. The difference was not statistically significant (*p* = 0.5) (Table 3).

Postoperative complications included seroma formation in 6 (10%) cases per group and bleeding in 6 (10%) per group. Facial nerve injury occurred in 9 (15%) patients in Group 2 and none in Group 1. Transient facial nerve palsy was observed in 9 (15%) cases in Group 2 only, with 3 (5%) progressing to permanent palsy, reflecting increased nerve vulnerability with the more extensive superficial approach (Table 4).

In terms of recurrence, 1 (1.7%) case was reported in Group 1, occurring 18 months after surgery, while 3 (5%) cases were observed in Group 2, with a mean time to recurrence of 15.5 ± 4.0 months. These findings suggest that both surgical techniques provide comparable long-term tumor control, with no statistically significant difference in recurrence rates (*p* = 0.6) (Table 5).

## 4. DISCUSSION

Parotid gland surgery, particularly for benign neoplasms, has evolved significantly to prioritize both oncological safety and functional preservation. While superficial parotidectomy has long been the gold standard, emerging evidence supports more conservative methods like intracapsular parotidectomy for selected benign tumors.^12^ The rationale behind investigating ICP as an alternative to SP stems from a growing awareness of the morbidity associated with traditional parotid surgery. Transient or even permanent facial nerve palsy, Frey’s syndrome, and cosmetic deformities are common concerns associated with SP. ICP, by avoiding routine exposure of the facial nerve and preserving surrounding tissues, may mitigate these risks, making it especially appealing in an era of patient-centered, minimally invasive surgical care.^5,6^ This study aimed to compare intracapsular and superficial parotidectomy in patients with benign parotid tumors, focusing on surgical efficiency, intraoperative and postoperative complications, nerve function, and recurrence rates, thereby providing high-quality evidence to guide clinical decision-making. Both groups were comparable in terms of demographic and tumor characteristics, including age, gender, tumor size, symptom duration, and comorbidities. This comparability strengthens the internal validity of our study, ensuring that any differences in outcomes can be more confidently attributed to the surgical technique rather than confounding variables. Laccourreye et al.^13^ emphasized the importance of baseline equivalence when comparing outcomes of different parotidectomy techniques.

Operative outcomes favored the intracapsular approach, with significantly shorter operative time (68.5 ± 12.3 min vs. 88.3 ± 14.5 min; *p* = 0.01) and lower intraoperative blood loss (120 ± 55.3 ml vs. 170 ± 50.5 ml; *p* = 0.001). This is because ICP avoids the meticulous dissection and identification of the facial nerve trunk, resulting in a shorter, more efficient procedure. McGurk et al.^14^ reported that extracapsular and intracapsular approaches can significantly reduce operative time compared to traditional superficial parotidectomy. Histopathological analysis showed no significant differences in tumor type between groups, with pleomorphic adenoma being the most common. Complication rates were generally low; however, transient facial nerve palsy occurred in 9 cases (15%) exclusively in the SP group, including 3 cases (5%) that became permanent. Because ICP does not require formal identification or dissection of the facial nerve trunk, it inherently reduces the risk of nerve trauma. The higher rate of nerve palsy in SP is likely related to manipulation and retraction around the facial nerve during surgery. Rates of seroma (10%) and postoperative bleeding (10%) were similar across both groups. Zbaren et al.^15^ reported a 23% incidence of permanent partial facial nerve injury following initial enucleation and 14% after initial superficial parotidectomy, including cases with preexisting or resected facial nerve issues. Recurrence was infrequent, observed in 1 patient (1.7%) in the ICP group and 3 patients (5%) in the SP group, indicating no statistically significant difference in long-term tumor control.

Careful case selection and meticulous surgical techniques in ICP can achieve similar long-term control of benign tumors as SP, despite avoiding wide margins. The low recurrence in both groups supports the oncological adequacy of ICP for benign tumors. Zbaren et al.^15^ found that recurrence rates for pleomorphic adenomas were low and comparable between ICP and SP when tumors were small, mobile, and well-circumscribed. A meta-analysis by Lin et al.^16^ including 1641 patients across seven studies (1120 ECD-treated and 521 SP-treated patients) showed that extracapsular dissection significantly reduced the risk of transient facial nerve injury and Frey’s syndrome compared to superficial parotidectomy, while other outcomes were similar. These findings align with our study, which also found lower complication rates and no significant difference in recurrence with intracapsular parotidectomy. Additionally, our results highlight perioperative advantages such as shorter operative time and less blood loss—not covered in the meta-analysis, suggesting better surgical efficiency and reduced morbidity with the intracapsular approach. A study by Zanghì et al.^12^ compared ECD and superficial parotidectomy in 56 patients and reported a significantly lower total complication rate in the ECD group, although individual complications were not statistically different. This agrees with our findings, where transient facial nerve palsy occurred only in the SP group, and the ICP group showed fewer complications overall. Both studies reported comparable recurrence rates and highlighted the better aesthetic outcomes and reduced surgical morbidity associated with ECD. These consistent findings reinforce the safety and functional advantages of the intracapsular approach for benign parotid tumors.

A large retrospective study by Kadletz et al.^17^ involving 894 patients found that extracapsular dissection was associated with higher rates of positive margins (29.4% vs 10.2%), recurrence (7.2% vs 2.2%), and permanent facial palsy (2.2% vs 0.6%) compared to superficial parotidectomy (SP) (all *p* < 0.05). These findings disagree with our results, where no permanent facial palsy occurred in the ICP group, and recurrence was slightly higher in the SP group. The discrepancy may be due to differences in surgeon experience, tumor selection, or follow-up duration. Notably, the referenced study still confirmed that SP required significantly longer operative time (146 vs 94 minutes), which aligns with our findings of shorter surgery duration and reduced intraoperative blood loss in the ICP group, supporting the procedural efficiency of function-preserving techniques. Kato et al.^18^ compared 26 cases of ECD and 20 of superficial parotidectomy, which showed that ECD was associated with significantly shorter procedure time, anesthesia time, length of hospital stay, and lower hospital charges. These results are in agreement with our findings, where ECD demonstrated a significantly shorter operative time and reduced intraoperative blood loss. Both studies also reported comparable rates of postoperative complications, including facial nerve weakness and great auricular nerve dysesthesia, supporting the safety profile of ECD. The consistency in reduced surgical burden and resource utilization further highlights ECD as a cost-effective and time-efficient alternative to SP for benign parotid tumors.

Our study possesses several notable strengths that enhance its methodological rigor and clinical relevance. First, the randomized design effectively minimizes selection bias, thereby strengthening the internal validity of outcome comparisons between intracapsular parotidectomy and superficial parotidectomy. Additionally, the inclusion of a homogeneous patient cohort limited to benign parotid tumors with comparable demographic and tumor characteristics ensured a focused and reliable analysis of the surgical techniques without the confounding influence of malignancies. Another strength lies in the comprehensive collection of perioperative data, including operative time, intraoperative blood loss, and a detailed profile of complications. These meticulously recorded parameters provide valuable insights into the practical and safety aspects of both procedures.

## 5. STUDY LIMITATIONS 

While expanding the sample to 120 patients has improved statistical power, the follow-up period remains relatively short (two years), which limits the ability to detect late recurrences, particularly for pleomorphic adenomas, known to recur as late as 5–10 years postoperatively. The single-center nature of the trial may also limit generalizability, as outcomes may reflect the expertise and surgical preferences of a specific institution. Finally, the study did not incorporate validated patient-reported outcomes or quality-of-life assessments, which are increasingly recognized as essential components of surgical evaluation.

## 6. CLINICAL RECOMMENDATIONS 

Intracapsular parotidectomy is a safe and effective alternative to superficial parotidectomy for benign, well-circumscribed parotid tumors. It offers clear advantages in reducing operative time, blood loss, and facial nerve injury, without compromising tumor control. Therefore, it should be considered the preferred approach in appropriately selected cases, especially when preserving facial function and aesthetics is a priority.

## ACKNOWLEDGMENTS

The author(s) have no acknowledgements to declare.

## AUTHOR CONTRIBUTIONS

AA, SR, TA, AE, HA, ZF, ME: manuscript preparation, protocol, data collection and management, manuscript editing. HR, AF, MI, AB, MA, HF, EM, MA: Data acquisition, data analysis and management, manuscript editing. IM, MH, MM, MA, AG, MM, AL: Manuscript editing, project development, data analysis, and project development. All the authors have read and approved the manuscript.

## CONFLICT OF INTEREST

The authors declare that they have no conflict of interest.

## DISCLOSURE OF AI USE

All authors declare that no AI used in this work.

## DATA AVAILABILITY STATEMENT

The data that support the findings of this study are available on request from the corresponding author.

## FUNDING

The author(s) received no financial support for the research, authorship, and/or publication of this article.

## ETHICS APPROVAL

Ethical committee approval was received from the Institutional Review Board of Al-Azhar University (Registration no: 043/25).

## Figures and Tables

**Table 1.** Demographic and baseline characteristics of the studied participants.

Variables	Total (*N* = 120)	Group 1 (Intracapsular) (*N* = 60)	Group 2 (Superficial) (*N* = 60)	*p-*value
**Age (Years)**				
Mean ± SD	46.3 ± 9.5	39.1 ± 6.8	53.4 ± 5.6	0.001*
Range	25–65	25–48	44–65	
**Gender**				
Male	84 (70%)	36 (60%)	48 (80%)	0.3
Female	36 (30%)	24 (40%)	12 (20%)	
**Tumor size (cm)**				
Mean ± SD	2.8 ± 0.9	2.7 ± 0.8	2.9 ± 1.0	0.31
Range	1.5–4.5	1.5–4	1.8–4.5	
**Duration of symptoms (months)**				
Mean ± SD	15.2 ± 6.4	14.9 ± 5.8	15.5 ± 7.0	0.72
Range	6–30	6–28	7–30	
**Comorbidities**				
DM	36 (30%)	15 (25%)	21 (35%)	0.3
HTN	27 (22.5%)	6 (10%)	21 (35%)	
**Pain**				
Present	45 (37.5%)	21 (35%)	24 (40%)	0.7

*indicates significant at the level of ≤ 0.05.

**Table 2.** Intraoperative and postoperative outcomes of the studied participants.

Variables	Total (*N* = 120)	Group 1 (Intracapsular) (*N* = 60)	Group 2 (Superficial) (*N* = 60)	*p-*value
**Operative time (Minutes)**				
Mean ± SD	78.4 ± 15.2	68.5 ± 12.3	88.3 ± 14.5	0.01*
Range	50–120	50–90	70–120	
**Blood loss (mL)**				
Mean ± SD	145 ± 56.2	120 ± 55.3	170 ± 50.5	0.02*
Range	50–300	50–200	100–300	

*indicates significant at the level of ≤ 0.05.

**Table 3.** Histopathological findings of the studied participants.

Variables	Total (*N* = 120)	Group 1 (Intracapsular) (*N* = 60)	Group 2 (Superficial) (*N* = 60)	*p-*value
Pleomorphic	78 (65%)	42 (70%)	36 (60%)	0.5
Warthin tumor	42 (35%)	18 (30%)	24 (40%)	

**Table 4.** Complications among the studied participants.

Variables	Total (*N* = 120)	Group 1 (Intracapsular) (*N* = 60)	Group 2 (Superficial) (*N* = 60)	*p-*value
Seroma	12 (10%)	6 (10%)	6 (10%)	0.3
Bleeding	12 (10%)	6 (10%)	6 (10%)	
Nerve injury	9 (7.5%)	0 (0%)	9 (15%)	
Transient facial nerve palsy	9 (7.5%)	0 (0%)	9 (15%)	
Permanent facial nerve palsy	3 (2.5%)	0 (0%)	3 (5%)	

**Table 5.** Recurrence and long-term outcomes among the studied participants.

Variables	Total (*N* = 120)	Group 1 (Intracapsular) (*N* = 60)	Group 2 (Superficial) (*N* = 60)	*p-*value
**Tumor recurrence rate (%)**				
Yes	4 (3.3%)	1 (1.7%)	3 (5%)	0.6
No	116 (96.7%)	59 (98.3%)	57 (95%)	
